# iSuc-ChiDT: a computational method for identifying succinylation sites using statistical difference table encoding and the chi-square decision table classifier

**DOI:** 10.1186/s13040-022-00290-1

**Published:** 2022-02-10

**Authors:** Ying Zeng, Yuan Chen, Zheming Yuan

**Affiliations:** 1 School of Computer and Communication, Hunan Institute of Engineering, Xiangtan, 411104 Hunan China; 2 Hunan Engineering & Technology Research Center for Agricultural Big Data Analysis & Decision-making, Hunan Agricultural University, Changsha, 410128 Hunan China

**Keywords:** Succinylation site, Chi-square statistical difference table, ChiDT, Imbalanced dataset, Feature selection

## Abstract

**Background:**

Lysine succinylation is a type of protein post-translational modification which is widely involved in cell differentiation, cell metabolism and other important physiological activities. To study the molecular mechanism of succinylation in depth, succinylation sites need to be accurately identified, and because experimental approaches are costly and time-consuming, there is a great demand for reliable computational methods. Feature extraction is a key step in building succinylation site prediction models, and the development of effective new features improves predictive accuracy. Because the number of false succinylation sites far exceeds that of true sites, traditional classifiers perform poorly, and designing a classifier to effectively handle highly imbalanced datasets has always been a challenge.

**Results:**

A new computational method, iSuc-ChiDT, is proposed to identify succinylation sites in proteins. In iSuc-ChiDT, chi-square statistical difference table encoding is developed to extract positional features, and has a higher predictive accuracy and fewer features compared to common position-based encoding schemes such as binary encoding and physicochemical property encoding. Single amino acid and undirected pair-coupled amino acid composition features are supplemented to improve the fault tolerance for residue insertions and deletions. After feature selection by Chi-MIC-share algorithm, the chi-square decision table (ChiDT) classifier is constructed for imbalanced classification. With a training set of 4748:50,551(true: false sites), ChiDT clearly outperforms traditional classifiers in predictive accuracy, and runs fast. Using an independent testing set of experimentally identified succinylation sites, iSuc-ChiDT achieves a sensitivity of 70.47%, a specificity of 66.27%, a Matthews correlation coefficient of 0.205, and a global accuracy index *Q*^9^ of 0.683, showing a significant improvement in sensitivity and overall accuracy compared to PSuccE, Success, SuccinSite, and other existing succinylation site predictors.

**Conclusions:**

iSuc-ChiDT shows great promise in predicting succinylation sites and is expected to facilitate further experimental investigation of protein succinylation.

**Supplementary Information:**

The online version contains supplementary material available at 10.1186/s13040-022-00290-1.

## Background

Protein post-translational modifications (PTMs) regulate cellular physiology and significantly increase protein diversity and complexity. Lysine succinylation is an evolutionarily conserved PTM present in both prokaryotic and eukaryotic cells where a succinyl group is covalently bonded to specific lysine residues by enzymatic or non-enzymatic processes [1, 2]. Succinylation can promote remarkable changes in protein structure and function, and plays a role in many diseases, such as tuberculosis [3], allergic dermatitis [4], and inflammation [5]. Therefore, elucidating the molecular mechanism of succinylation will provide valuable information for both biomedical research and drug development.

Accurate identification of succinylation sites is critical to succinylation research, and because experimental methods are costly and time-consuming, and have been unable to keep up with the exponential growth of the number of sequenced proteins, efficient in silico methods are in great demand. To date, many predictors for identifying succinylation sites have been developed, such as SucPred [6], SuccinSite [7], pSuc-Lys [8], PSuccE [9] and so on, but with their limited overall accuracy and poor sensitivity, numerous true succinylation sites remain undetected. Actually, what interested us more is the information on true succinylation sites. Therefore, it is necessary to further improve predictive accuracy, especially sensitivity. Two key components, feature extraction and classifier construction, can greatly affect the accuracy of a computational method.

Commonly used features include positional features [7, 9–11], sequence composition [7–11], evolutionary information [12–14], and protein secondary structure [13–15]. Positional information of amino acids is basic but important to a protein sequence. While binary encoding [7, 9] is the most intuitive method to extract positional features, the feature matrix is very sparse. The binary encodings are the same for the same residue at different positions, and so it cannot reflect positional differences. Physicochemical property encoding [7, 9, 11] is another position-based amino acid encoding scheme that is frequently used. The AAindex [16] database records 531 physicochemical properties of 20 standard amino acids. Since it is not known in advance which physicochemical properties are related to classification, physicochemical property encoding means each position needs to be represented by 531 physicochemical properties, resulting in many irrelevant and redundant features.

Traditional classifiers including support vector machine (SVM) [6, 9–11, 13], random forest (RF) [7, 8] and decision tree [12, 15] have been applied in succinylation site prediction. The number of false succinylation sites (non-succinylated lysine residues) far exceeds that of true sites, for example, the dataset from Hasan et al. [7] contains 5004/53524 true/false succinylation sites (a ratio of positive to negative samples of about 1:10). Training any traditional classifier with such highly imbalanced datasets could strongly bias classification results [17], and the large number of training samples would make the training time of some classifiers (e.g*.* SVM) unbearable. To address this, some methods (e.g. SucPred, SuccinSite) balanced the class distribution by under-sampling the negative samples, but this might lead to the loss of some potential classification information due to the mass discarding of negative samples; some methods (e.g. pSuc-Lys, PSuccE) designed classifier ensemble algorithms, however, they were still integrated results of several individual classifiers trained with a balanced subset where positive samples were repeatedly used.

**Fig. 1:**
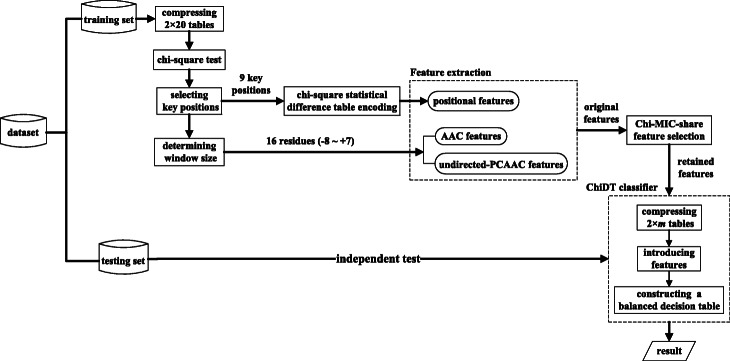
Flow chart of iSuc-ChiDT

Based on a highly imbalanced dataset, we developed an efficient approach called iSuc-ChiDT for predicting succinylation sites. Firstly, the 2 × 20 contingency table of each position was compressed based on local chi-square tests, and then the 9 key positions and a window size of 16 residues were determined. Next, chi-square statistical difference table encoding was used to characterize the 9 key positions, and amino acid composition (AAC) and undirected pair-coupled amino acid composition (undirected-PCAAC) features were incorporated. After applying the Chi-MIC-share [18] algorithm for feature selection, the ChiDT classifier was finally designed to achieve imbalanced classification. The flow chart of our method is shown in Fig. 1.

## Methods

### Datasets

From Uni-ProtKB/Swiss-Prot [19] database and NCBI protein sequence database [20], Ning et al. [9] obtained 2322 succinylated proteins with 5009 experimentally verified lysine succinylation sites by applying a 30% homology-reducing screening procedure with CD-HIT [21]. Then 124 succinylated proteins were randomly selected to build an independent testing set, and the remaining 2198 succinylated proteins were used as a training set. In this study, we used the same training and independent testing dataset as in Ning et al*.*, which were freely available via the web link [22]. Our training set, namely Tr_data, contains 4748/50,551 true/false succinylation sites; and our testing set, namely Te_data, contains 254/2977 true/false succinylation sites.

Each true/false succinylation site was represented by a sequence fragment with an initial length of 51 amino acid residues, where the candidate site (lysine residue) was at the central position 0, and the upstream positions were successively labeled as − 1, − 2, …, − 25, and the downstream positions labeled 1, 2, …, 25. If the number of up- or downstream residues of the candidate site was less than 25, amino acids were created through mirror extension to make up the difference [8]. For example, the original sequence of the succinylated protein “SP-P0ABS8” is “ML**K**NLAKLDQTEMDKVNVDLAAAGVAFKE …” . The first lysine (*K*) is the candidate site and therefore the sequence fragment generated by mirror extension is “KFAVGAAALDVNVKDMETQDLKAML**K**NLAKLDQTEMDKVNVDLAAAGVAFK”. All sequence samples contain only the 20 standard amino acids.

### Compression for the 2 × 20 contingency table of each position

The maximal information coefficient (MIC) [23] is a novel measure proposed to capture dependences between paired variables. The MIC score ranges from 0 to 1, and only approaches 0 if two variables are statistically independent. To calculate the MIC score of the paired variables *x* and *y*, the ApproxMaxMI [23] algorithm sets the *n*_*x*_ × *n*_*y*_< B(*n*), where B(*n*) = *n*^0.6^ is the maximal grid size restriction, and *n* is the sample size, and *n*_*x*_, *n*_*y*_ are the number of partition bins on *x* and *y*, respectively. The MIC score for two independent variables calculated by ApproxMaxMI depends on the ratio between B(*n*) and *n* [24], and it is close to 0 only when *n* approaches infinity. For two independent variables under finite samples (especially for small sample size), ApproxMaxMI leads to a large deviation between the calculated MIC score and 0, meaning that the MIC will capture false associations. To address this drawback, Chen et al*.* [25] proposed an improved algorithm, ChiMIC [25], which uses local chi-square test to determinate optimal bin size for the calculating of MIC score. For two independent variables with 100 sample points, ApproxMaxMI tends to fall into the maximal grid size (100^0.6^ ≈ 16), and the corresponding grid partition is a 2 × 8 grid, and the MIC score is 0.24. With ChiMIC, the MIC score is only 0.06, and the corresponding grid partition is a 2 × 2 or 2 × 3 grid. This shows that the grid partition searched by ChiMIC is more reasonable and that compressing a 2 × 8 grid into a 2 × 2 or 2 × 3 grid is wise.

**Fig. 2:**
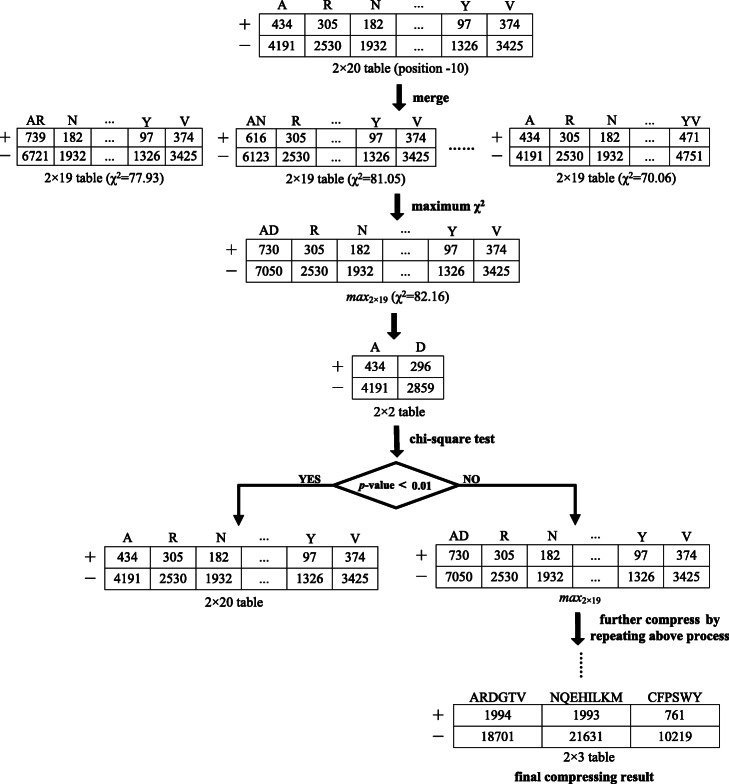
Illustration of compression procedure (position − 10 in Tr_data)

Similarly, for each position in succinylation site-containing sequences, we can construct a 2 × 20 contingency table by respectively counting the occurrence frequencies of the 20 standard amino acids in the positive and negative samples*.* For instance, Fig. 2 gives the 2 × 20 table of position − 10 in Tr_data. What we need to investigate is whether the 2 × 20 Table (2 × 20 grid) is reasonable, and could it be compressed into a 2 × 10, or even a 2 × 2 table? A similar attempt was made in donor splice site prediction. For each position in donor site-containing sequences, a 2 × 4 contingency table can be built by counting the frequencies of 4 bases in the positive and negative samples. Following on from ChiMIC, Zeng et al*.* [26] compressed the 2 × 4 table of each position into a 2 × *l* (2 ≤ *l* ≤ 4) table using local chi-square test, and developed a high-performance approach to predict donor splice sites based on this compression strategy.

Encouraged by the successful application of the compression strategy on nucleotide sequences, we applied it to protein sequences. For the 2 × 20 contingency table for each position in succinylation site-containing sequences, the compression procedure is described below.

Step 1: Set the initial value of *r* (*r* is an integer) to 20.

Step 2: The 2 × *r* contingency table is compressed by merging two columns corresponding to two different residues, and some 2 × (*r*-1) contingency tables are obtained, then select a 2 × (*r*-1) contingency table with the maximum chi-square value, denoted as *max*_2 × (*r*-1)_.

Step 3: A local 2 × 2 contingency table is constructed based on the merged residues in *max*_2 × (*r*-1)_ and perform a chi-square test. If the *p*-value is lower than a given threshold, *max*_2 × (*r*-1)_ is unreasonable and will be backtracked to the 2 × *r* contingency table and the compression procedure is terminated. If the *p*-value is greater than a given threshold, *max*_2 × (*r*-1)_ is reasonable, and a further compression of *max*_2 × (*r*-1)_ is attempted following these steps: 1) set *r* = *r*-1; 2) if *r* ≥ 3, repeat Step 2 ~ 3; otherwise, terminate compression.

Tc − 10 in Tr_data as an example, its 2 × 20 contingency table was finally compressed into a 2 × 3 table (Fig. 2). The 20 original status values of position − 10 were therefore turned into 3 status values, i.e., “ARDGTV”, “NQEHILKM” and “CFPSWY”, where, “ARDGTV” indicated that A, R, D, G, T, V at position − 10 were regarded as the same status value, and the others were similar.

### Key positions selection and window size determination

**Fig. 3:**
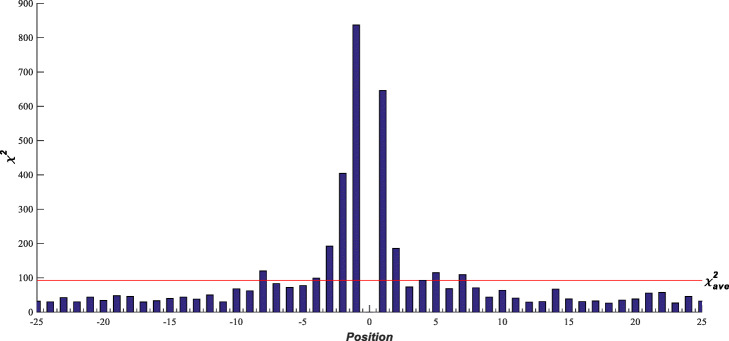
Chi-square values for different positions in Tr_data

For each position in the sequences with 51 residues, a 2 × *r* (2 ≤ *r* ≤ 20) contingency table can be obtained after compression based on the training set. A chi-square test was then performed on the 2 × *r* contingency table and the corresponding chi-square value was calculated. Higher chi-square values indicate that the corresponding positions are more important for discriminating positives from negatives. Figure 3 shows the chi-square values of 50 positions (− 25 ~ + 25, excluding position 0) in Tr_data, and the chi-square tests of all the positions are significant. We calculate the average of the chi-square values of all the positions, denoted as ${\chi}_{ave}^2$, then set ${\chi}_{ave}^2$ as the threshold to select key positions. The chi-square values of positions − 8, − 4 ~ − 1, 1, 2, 5, 7 are above ${\chi}_{ave}^2=92.797$ (see the red line in Fig. 3), therefore these 9 positions are regarded as the key positions. Furthermore, the contiguous 16 residues (positions − 8 ~ + 7) are determined as the window size.

### Positional feature extraction

A new position-based amino acid encoding scheme, chi-square statistical difference table encoding, was developed for position characterization. For the 9 key positions in each sequence sample, we extracted 9 positional features based on chi-square statistical difference table encoding, denoted as *P*_− 8_, *P*_− 4_, *P*_− 3_, *P*_− 2_, *P*_− 1_, *P*_1_, *P*_2_, *P*_5_ and *P*_7_ respectively, where, *P*_− 8_ represents the positional feature of position − 8, *P*_− 4_ represents the positional feature of position − 4, and so forth.

In the training set, the occurrence frequencies of the 20 standard amino acids were counted at the *i*^th^ (*i* = 1, 2, …, 9) position in the positive and negative samples, and then a 2 × 20 contingency table was built (Table 1).

**Table 1:** Frequency distribution of amino acids at the *i*^th^ position

Sample	Amino acid residue						Total
	1	2	…	*j*	…	20	
Positive	${f}_{i,1}^{+}$	${f}_{i,2}^{+}$	…	${f}_{i,j}^{+}$	…	${f}_{i,20}^{+}$	${f}_i^{+}$
Negative	${f}_{i,1}^{-}$	${f}_{i,2}^{-}$	…	${f}_{i,j}^{-}$	…	${f}_{i,20}^{-}$	${f}_i^{-}$
Total	*f*_*i*, 1_	*f*_*i*, 2_	…	*f*_*i*, *j*_	…	*f*_*i*, 20_	*N*

In Table 1, ${f}_{i,j}^{+}$ represents the frequency of the *j*^th^ (*j* = 1,2, …,20) residue at the *i*^th^ position in the positive samples, ${f}_{i,j}^{-}$ represents the corresponding frequencies in the negative samples, ${f}_i^{+}$ and ${f}_i^{-}$ represent the total number of positive and negative samples, and *N* represents the total number of samples. The chi-square value corresponding to the *i*^th^ position is calculated by:

1 $$ {\chi}^2=\frac{N^2}{f_i^{+}\times {f}_i^{-}}\left[\sum \limits_{j=1}^{20}\frac{{f_{i,j}^{+}}^2}{f_{i,j}}-\frac{{f_i^{+}}^2}{N}\right] $$

If a new training sample is added, and the *j*^th^ residue appears at the *i*^th^ position, first assume this sample is positive, replace ${f}_{i,j}^{+}$ with ${f}_{i,j}^{+}+1$, and calculate a chi-square value ${\chi}_{i,j}^{2+}$ using formula (1); then assume this sample is negative, replace ${f}_{i,j}^{-}$ with ${f}_{i,j}^{-}+1$, and calculate a chi-square value ${\chi}_{i,j}^{2-}$ using formula (1). The score for the chi-square statistical difference table with the *j*^th^ residue at the *i*^th^ position is defined as:

2 $$ \Delta {\chi}_{i,j}^2={\chi}_{i,j}^{2+}-{\chi}_{i,j}^{2-} $$

Next, build a 20 × 9 chi-square statistical difference table (Table 2). Table 2 gives the scores of the various amino acid residues at each position. If the *j*^th^ residue appears at the *i*^th^ position, the *i*^th^ positional feature will be assigned a value of $\Delta {\chi}_{i,j}^2$. Table S1 (Additional file 1) shows the 20 × 9 chi-square statistical difference table constructed based on 9 key positions in Tr_data.

**Table 2:** 20 × 9 chi-square statistical difference table

Amino acid residue	Position				
	1	…	*i*	…	9
1	$\Delta {\chi}_{1,1}^2$	…	$\Delta {\chi}_{i,1}^2$	…	$\Delta {\chi}_{9,1}^2$
…	…	…	…	…	…
*j*	$\Delta {\chi}_{1,j}^2$	…	$\Delta {\chi}_{i,j}^2$	…	$\Delta {\chi}_{9,j}^2$
…	...	…	…	…	…
20	$\Delta {\chi}_{1,20}^2$	…	$\Delta {\chi}_{i,20}^2$	…	$\Delta {\chi}_{9,20}^2$

### Compositional feature extraction

Despite positional features could distinguish highly similar positive and negative samples, they lack fault tolerance when there are residue insertions and deletions in protein sequences. Compositional features can capture the context correlation while reflecting sequence composition, and they are more fault-tolerant. Giving an example as follows:

**Table:** 

Position:	1	2	3	4	5	6	7	8	9	10	11	12
Original sequence segment:	*R*	*F*	L	A	*N*	Y	V	T	K	A	G	K
Mutated sequence segment:	*R*	*F*	L	**E**	A	*N*	Y	V	T	K	A	G

The mutated sequence segment is caused by the insertion of residue E at position 4 of the original sequence segment. Obviously, the positional features changed a lot after residue inserting, but the sequence components changed little. Therefore, we supplement compositional features in hope of improving the algorithm’s robustness to residue insertions and deletions.

For each sequence sample with a window size of 16 residues, 230 compositional features were extracted, including 20 AAC features and 210 undirected-PCAAC features.

The AAC features are defined as the occurrence frequencies of the 20 standard amino acids in the sequence, respectively denoted as *f*_*A*_, *f*_*R*_, …, *f*_*V*_, where, *f*_*A*_ represents the frequency of alanine (*A*), *f*_*R*_ represents the frequency of arginine (*R*), and so forth.

The individual amino acid components are independent of each other, so the AAC features cannot reflect any correlation between amino acids. The pair-coupled amino acid composition [27] (PCAAC) features are composed of the occurrence frequencies of pairwise coupling between two adjacent residues, which can reflect both sequence components and the most preliminary association effect. To reduce feature dimension and solve the sparse problem of feature matrix, we assume that the pairwise coupling has no direction. For example, A-R coupling is treated the same as R-A coupling, and the corresponding pair-coupled component will be expressed by either *f*_*AR*_ or *f*_*RA*_, where *f*_*AR*_ (*f*_*RA*_) is the sum of AR pair occurrence frequency and RA pair occurrence frequency found in a sequence.

### Feature selection based on chi-MIC-share

In order to eliminate irrelevant features and redundant features in the original feature set and reduce the number of features, we decided to perform feature selection. Minimum redundancy maximum relevance (mRMR) [28] is a popular feature selection method. However, relevance measure and redundancy measure in mRMR are not comparable, mRMR only gives the order of feature introduction and it is time-consuming to perform cross-validation in training sets to get the optimal feature subset. To address this, Li et al. [18] used ChiMIC as the unified measure of relevance and redundancy, and designed a redundancy sharing strategy to propose a novel feature selection method, Chi-MIC-share. Here, we applied Chi-MIC-share for feature selection.

Given an original feature set *Ω* = {*X*_1_, *X*_2_, …, *X*_*i*_, …, *X*_*n*_}, *|Ω|* is the number of elements in *Ω*, and *|Ω|* = *n*. If the introduced feature set is represented by *S*, the complement of *S* is represented as *Ω*_*S*_ = *Ω*-*S.* Denoting the response variable as *Y*, the Chi-MIC-share algorithm is described as follows.

For an introduced feature *X*_*i*_ in *S*, the score after redundancy sharing is calculated by:

3 $$ \mathrm{Chi}\hbox{-} \mathrm{MIC}\hbox{-} \mathrm{share}\left({X}_i\right)=\sum \limits_{X_j\in S}\frac{\mathrm{Chi}\hbox{-} \mathrm{MIC}\left({X}_i;Y\right)}{\mathrm{Chi}\hbox{-} \mathrm{MIC}\left({X}_i;{X}_j\right)} $$

The total score of all features in *S* after redundancy sharing is:

4 $$ \mathrm{Chi}\hbox{-} \mathrm{MIC}\hbox{-} \mathrm{share}(S)=\sum \limits_{X_i\in S}\frac{\mathrm{Chi}\hbox{-} \mathrm{MIC}\left({X}_i;Y\right)}{\sum \limits_{X_j\in S}\mathrm{Chi}\hbox{-} \mathrm{MIC}\left({X}_i;{X}_j\right)} $$

If the next introduced feature is *X*_*next*_, set *E* = *S+*{*X*_*next*_}, then *|E|* = *|S|* + 1. The criterion for introducing the next optimal feature is:

5 $$ \underset{X_{next}\in {\varOmega}_S}{\mathit{\max}}\left[\mathrm{Chi}\hbox{-} \mathrm{MIC}\hbox{-} \mathrm{share}(E)\right]=\sum \limits_{X_i\in E}\frac{\mathrm{Chi}\hbox{-} \mathrm{MIC}\left({X}_i;Y\right)}{\sum \limits_{X_j\in E}\mathrm{Chi}\hbox{-} \mathrm{MIC}\left({X}_i;{X}_j\right)} $$

If a new introduced feature no longer makes the total Chi-MIC-share score increase, this feature will be discarded and feature selection will be automatically terminated. Thus, the criterion for terminating feature introduction is:

6 $$ \mathrm{Chi}\hbox{-} \mathrm{MIC}\hbox{-} \mathrm{share}(E)\le \mathrm{Chi}\hbox{-} \mathrm{MIC}\hbox{-} \mathrm{share}(S) $$

Furthermore, feature introduction can be forced to terminate according to the following criterion:

7 $$ \frac{\mathrm{Chi}\hbox{-} \mathrm{MIC}\hbox{-} \mathrm{share}(E)-\mathrm{Chi}\hbox{-} \mathrm{MIC}\hbox{-} \mathrm{share}(S)}{\mathrm{Chi}\hbox{-} \mathrm{MIC}\hbox{-} \mathrm{share}(S)}\le 0.01 $$

### Classifier construction

To efficiently achieve imbalanced classification, a classifier called ChiDT is designed as follows.

#### Compress the 2 × *m* contingency table of each retained feature

For each feature retained by the Chi-MIC-share feature selection, its 2 × *m* contingency table (*m* is the number of original status values of the feature) was compressed according to the previously described procedure to obtain a 2 × *r* contingency table (*r* is the number of new status values of the feature, 2 ≤ *r ≤ m*). During the compression process, since the status values of each retained feature are continuous, only adjacent status values could be merged together.

#### Introduce the retained features one by one

Supposing the proportion of the *k*^th^ class samples in sample set *D* is *p*_*k*_ (*k* = 1, 2), the information entropy of *D* is defined as:

8 $$ H(D)=-\sum \limits_{k=1}^2{p}_k{\log}_2{p}_k $$

Given a Chi-MIC-share retained feature *X*_*i*_, supposing it has *r* new status values as {*s*_1_, *s*_2_, …, *s*_*j*_, …, *s*_*r*_} after compressing, then the information gain that *X*_*i*_ brings for *D* can be calculated by:

9 $$ Gain\left(D,{X}_i\right)=H(D)-\sum \limits_{j=1}^r\frac{\mid {D}^j\mid }{\mid D\mid }H\left({D}^j\right) $$

where *D*^*j*^ represents the samples in *D* whose *X*_*i*_ takes the status value as *s*_*j*_ (1 ≤ *j* ≤ *r*), while *H* (*D*^*j*^) is the information entropy of *D*^*j*^.

From the features whose information gains are above the average, pick out the one with the highest gain ratio to be the first introduced feature. Here, the gain ratio of *X*_*i*_ is defined as:

10 $$ GainRatio\left(D,{X}_i\right)=\frac{Gain\left(D,{X}_i\right)}{IV\left({X}_i\right)} $$

where

11 $$ IV\left({X}_i\right)=-\sum \limits_{j=1}^r\frac{\left|{D}^j\right|}{\left|D\right|}{\log}_2\frac{\left|{D}^j\right|}{\left|D\right|} $$

and *IV* (*X*_*i*_) is the intrinsic value of *X*_*i*_.

Next, the remaining features are introduced one by one with the following steps.

Step 1: Under the conditions in which the introduced features have existed, the 2 × *r* contingency table of each remaining feature is further compressed. If the *r* columns of the 2 × *r* contingency table are compressed into one column, the remaining feature cannot be introduced; if the *r* columns are not compressed into one column, the remaining feature will be considered as a candidate feature to be introduced.

Step 2: Calculate the information gain of every candidate feature. From the candidate features whose information gains are above the average, the one with the highest gain ratio is selected to be the next introduced feature.

Step 3: Repeat Step 1 ~ 2 until no further features can be introduced.

After this, the introduced features with their status values generate various rules. Taking Tr_data as an example, 10 Chi-MIC-share retained features were finally introduced and 137 rules were generated (see Additional file 2: Table S2).

#### Construct a balanced decision table for decision-making

We counted the number of positive and negative training samples conforming to each rule then constructed a 2 × 137 imbalanced decision table (Table 3).

**Table 3:** Imbalanced decision table

Sample	Rule^*^			Total
	(*P*_−1_ = − 2.028) ˄ (− 0.907 ≤ *P*_2_ ≤ 0.501) ˄ (− 0.715 ≤ *P*_− 8_ ≤ 0.066)	…	(*P*_− 1_ = 1.839) ˄ (*P*_1_ = 2.060)	
Positive	23	…	32	4748
Negative	2907	…	83	50,551

*For instance, “(*P*_−1_ = −2.028)˄(−0.907 ≤ *P*_2_ ≤ 0.501)˄(− 0.715 ≤ *P*_− 8_ ≤ 0.066)” represents *P*_− 1_ taking a value of − 2.028 and *P*_2_ ranging from − 0.907 to 0.501 and *P*_− 8_ ranging from − 0.715 to 0.066, where, “˄” denotes the logical conjunction

The number of negative samples far exceeds the positives. To resolve the imbalanced classification problem, based on cost-sensitive learning [29], we adjust the decision weight of negative samples in each column of the imbalanced decision table, by multiplying the number of negative samples in each column of Table 3 by *θ*, where *θ* is defined as the ratio of the total number of positive and negative training samples, here, *θ* = 4748/50551. Then, a 2 × 137 balanced decision table is obtained (Table 4).

**Table 4:** Balanced decision table

Sample	Rule			Total
	(*P*_−1_ = − 2.028) ˄ (− 0.907 ≤ *P*_2_ ≤ 0.501) ˄ (− 0.715 ≤ *P*_− 8_ ≤ 0.066)	…	(*P*_− 1_ = 1.839) ˄ (*P*_1_ = 2.060)	
Positive	23	…	32	4748
Negative	273.04	…	7.80	4748

Then the balanced decision table (Table 4) is used for decision-making. Suppose that a testing sample meets the rule “(*P*_-1_=-2.028) ˄ (-0.907≤*P*_2_≤0.501) ˄ (-0.715≤*P*_-8_≤0.066)”. First, we assume that it is positive and replace 23 with 23 + 1, then calculate the corresponding chi-square value ${\chi}_{+}^2$. We then assume that it is negative and replace 273.04 with 273.04 + 1, then calculate the corresponding chi-square value ${\chi}_{-}^2$. If ${\chi}_{+}^2>{\chi}_{-}^2$, the testing sample is predicted to be positive, if not, it is negative.

### Performance evaluation

Sensitivity (SN), specificity (SP) and Matthews correlation coefficient (MCC) as the common indexes for evaluating binary classification are defined as follows:

12 $$ SN=\frac{TP}{TP+ FN} $$

13 $$ SP=\frac{TN}{TN+ FP} $$

14 $$ MCC=\frac{TP\times TN- FN\times FP}{\sqrt{\left( TP+ FN\right)\times \left( TP+ FP\right)\times \left( TN+ FP\right)\times \left( TN+ FN\right)}} $$

Here, TP, FP, TN, and FN denote the numbers of true positives, false positives, true negatives, and false negatives. MCC is a balanced statistical index that considers SN and SP, but it is sensitive to class distribution in a testing set. As shown in Table 5, when a prediction model has a SN of 93% and a SP of 95%, as the imbalance degree of the testing set grows, the MCC value declines. This shows that a low MCC value does not always indicate poor prediction performance as it may be caused by a highly imbalanced testing set.

**Table 5:** Various evaluation indexes on different ratios of positives to negatives

Positives/Negatives^*^	SN (%)	SP (%)	MCC	*Q*^9^
100/100	93.00	95.00	0.880	0.939
100/1000	93.00	95.00	0.752	0.939
100/10000	93.00	95.00	0.371	0.939

*positive testing sample size/negative testing sample size

The content-balancing accuracy index *Q*^9^ [30] is independent of the class distribution of the dataset and has been widely used to evaluate performance of many prediction programs including gene-finding, splice site prediction and protein secondary structure prediction [31–33]. As Table 5 shown, the value of *Q*^9^ remains unchanged across different ratios of positives to negatives. In this study, we introduced *Q*^9^ as the measure of global accuracy to evaluate the prediction performance of models in case of an imbalanced testing set. *Q*^9^ is defined as:

15 $$ {Q}^9=\left(1+{q}^9\right)/2 $$

where

$$ {q}^9=\left\{\begin{array}{l}\left(\mathrm{TN}\hbox{-} \mathrm{FP}\right)/\left(\mathrm{TN}+\mathrm{FP}\right),\kern10.5em \mathrm{if}\kern0.5em \mathrm{TP}+\mathrm{FN}=0\\ {}\left(\mathrm{TP}\hbox{-} \mathrm{FN}\right)/\left(\mathrm{TP}+\mathrm{FN}\right),\kern10.5em \mathrm{if}\kern0.5em \mathrm{TN}+\mathrm{FP}=0\\ {}1\hbox{-} \sqrt{2}\sqrt{{\left[ FN/\left( TP+ FN\right)\right]}^2+{\left[ FP/\left( TN+ FP\right)\right]}^2},\mathrm{if}\kern0.5em \mathrm{TP}+\mathrm{FN}\ne 0\ \mathrm{and}\ \mathrm{TN}+\mathrm{FP}\ne 0\end{array}\right. $$

The value of *Q*^9^ ranges from 0 to 1, and the larger the *Q*^9^ value, the better the prediction performance.

## Results and discussion

### Features retained by chi-MIC-share

**Fig. 4:**
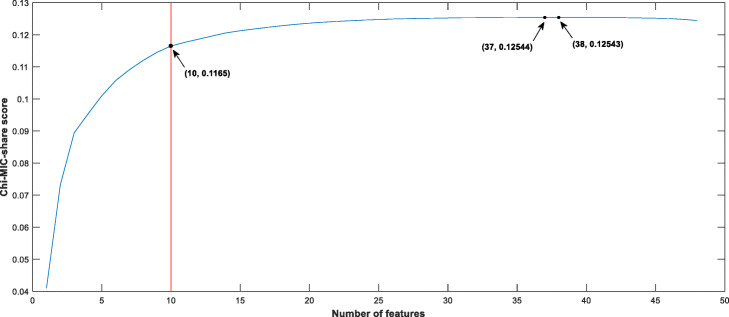
Chi-MIC-share scores after introduction of each feature. The red line represents the forced termination of feature introduction

Based on Tr_data, the Chi-MIC-share feature selection was performed on 239 original input features (9 positional features and 230 compositional features). As shown in Fig. 4, when the 37^th^ feature was introduced, the Chi-MIC-share score peaked (0.12544), after which is began to decline and feature selection was automatically terminated. To improve computational efficiency, forced termination criteria were adopted and 10 features were retained (see the red line in Fig. 4). Table 6 describes the retained features in detail. It can be seen that positional features contribute 80% of all the retained features, indicating that positional features have an important contribution to succinylation site prediction.

**Table 6:** Features retained by Chi-MIC-share

*No.*	Retained features	Type		*No.*	Retained features	Type
1	*P*_− 1_	position		6	*P*_7_	position
2	*P*_1_	position		7	*f*_*R*_	AAC
3	*P*_−2_	position		8	*f*_*K*_	AAC
4	*P*_5_	position		9	*P*_2_	position
5	*P*_−8_	position		10	*P*_−3_	position

*No*. denotes the order of feature introduction.

### Comparison of different classifiers

Based on the same input features (10 features retained by Chi-MIC-share), the ChiDT classifier was compared to traditional classifiers including RF, artificial neural network (ANN) and relaxed variable kernel density estimator (RVKDE) [34]. We choose RVKDE for the comparison because it delivers the same level of accuracy as SVM when the number of training samples exceeds 10,000, with a significantly lower average time complexity of *O* (*n*log*n*) [34], *n* denotes the number of training samples. RF and ANN classifiers were built with Weka 3.8.1 and the neural network toolbox in Matlab R2015a, respectively, and all parameters took the default values. The independent tests based on Tr_data and Te_data were performed for comparison (Table 7).

**Table 7:** Independent test accuracy for different classifiers based on the same input features

Classifier	SN (%)	SP (%)	MCC	*Q*^9^
RF	2.75	99.83	0.115	0.312
ANN	0	99.90	−0.009	0.293
RVKDE	9.84	97.25	0.106	0.362
**ChiDT**	**70.47**	**66.27**	**0.205**	**0.683**

The results show that: 1) ChiDT achieved a significantly higher predictive accuracy and effectively realized imbalanced classification. When the training set was imbalanced (4748 positives/50,551 negatives), the prediction results of RF, ANN and RVKDE were biased to negative samples, resulting in poor sensitivities (SNs < 10%). With ANN, while specificity was up to 99.9%, sensitivity was equal to 0. This meant that all positive samples were predicted to be negative and thus the global accuracy of ANN was the lowest (*Q*^9^ = 0.293). ChiDT built a balanced decision table through weighted correction strategy to perform imbalanced classification and obtained the highest accuracy (*Q*^9^ = 0.683). 2) ChiDT has a satisfactory calculation speed and can be applied to large samples. All simulations were run on an Intel Core i5-3320M 2.6 GHz/8 GB RAM system, and the elapsed time of ChiDT and RVKDE were 17 s and 18 min, respectively. ChiDT’s high speed is achieved because there is no need for parameter optimization.

### Comparison of different position-based encoding schemes

For the 9 key positions in Tr_data (here using 4748 positive samples and 4748 negative samples), we respectively used binary encoding, physicochemical property encoding (including 531 physicochemical properties [7] and 10 physicochemical properties [9] for encoding) and chi-square statistical difference table encoding to extract positional features, and then employed ChiDT classifier for prediction. The results of 5-fold cross validation showed that chi-square statistical difference table encoding achieved the highest predictive accuracy and the fewest features (Table 8).

**Table 8:** 5-fold cross accuracy for different encoding schemes based on ChiDT classifier

Encoding scheme	Feature dimension	SN (%)	SP (%)	MCC	Q^9^
Binary	180	63.20	62.41	0.258	0.623
Physicochemical properties(531)	4779	58.86	60.39	0.188	0.593
Physicochemical properties(10)	90	59.77	62.59	0.225	0.607
**Chi-square statistical difference table**	**9**	**65.94**	**62.91**	**0.290**	**0.641**

Binary encoding means that each position is represented by 20 0/1-features and the corresponding feature matrix is therefore very sparse. When using binary encoding scheme, the encodings of the same residue at different positions are the same, which does not reflect positional difference, and for different residues at the same position, it does not reflect the degree of difference between residues. For example, the amino acid polarity indexes of residue S, T and R at the same position are 1.67, 1.66 and 52, respectively, indicating that the polarity difference between S and T is small, and between T and R is large, but the hamming distances of both S-T and S-R are equal to 2 when using binary encoding. As for physicochemical property encoding, when 531 amino acid indices in AAindex were all considered for sequence characterization, the number of features reached 531 × 9 = 4779 (Table 8), and a lot of irrelevant and redundant features would be seen. Ning et al [9] ranked 531 physicochemical properties according to their abilities to distinguish between true and false succinylation sites, then chose the top 10 physicochemical properties for sequence encoding, so that the feature dimension was greatly reduced. However, as shown in Table 8, no matter whether 531 or 10 physicochemical properties are used, the predictive accuracy is always lower than that of chi-square statistical difference table encoding.

Chi-square statistical difference table encoding reflects the difference of the same residue at different positions, as well as the degree of difference between different residues at the same position, thus, it could differentiate between the highly similar positive and negative samples. Another benefit of chi-square statistical difference table encoding is that it has a low feature dimension, low redundancy, and a non-sparse feature matrix.

### Comparison of different window sizes

Based on Tr_data and Te_data, independent tests were performed to compare the prediction performance of the determined window size (− 8 ~ + 7) with longer (e.g. -25 ~ + 25, − 15 ~ + 15) and shorter window sizes (e.g. -5 ~ + 5). Specifically, under each window size, we extracted 9 positional features based on statistical difference table encoding (7 positional features for the window size of − 5 ~ + 5, as it includes only 7 key positions) and 230 compositional features (including 20 AAC features and 210 undirected-PCAAC features), after Chi-MIC-share feature selection, incorporated ChiDT classifier for prediction. The results (Table 9) show that the proposed model with a window size of 16 residues (− 8 ~ + 7) can achieve higher independent test accuracy compared to other window sizes. This indicates that an overly long window size may introduce some irrelevant information, while too short a window may lead to insufficient information collection, both of which reduce predictive accuracy. This confirms that our window size determination is reliable.

**Table 9:** Independent test accuracy based on different window sizes

Window size	SN (%)	SP (%)	MCC	Q^9^
51(− 25 ~ + 25)	68.50	61.10	0.162	0.646
31(− 15 ~ + 15)	64.57	66.48	0.174	0.655
**16(−8 ~ + 7)**	**70.47**	**66.27**	**0.205**	**0.683**
11(−5 ~ + 5)	62.20	65.03	0.152	0.636

### Necessity of chi-MIC-share feature selection

Based on Tr_data and Te_data, the independent test results with or without Chi-MIC-share feature selection are shown in Table 10. They show that feature selection based on Chi-MIC-share can: 1) improve predictive accuracy, with the Q^9^ value improving from 0.663 to 0.683, and 2) reduce feature dimension and save computational time. After feature selection, the number of original input features was reduced from 239 to 10, and the elapse time of ChiDT was reduced by 95%. Therefore, it is necessary and beneficial to perform a Chi-MIC-share feature selection.

**Table 10:** Independent test accuracy with or without Chi-MIC-share

Feature selection	Feature dimension	SN (%)	SP (%)	MCC	*Q*^9^	Time (mm:ss)
No feature selection	239	70.08	62.95	0.182	0.663	06:14
Chi-MIC-share	10	70.47	66.27	0.205	0.683	00:17

### Comparison with existing methods

To further evaluate the performance of our method (iSuc-ChiDT), we compared it with existing succinylation site predictors, SucPred, iSuc-PseAAC [10], SuccFind [11], SuccinSite, iSuc-PseOpt [35], pSuc-Lys, Success [13] and PSuccE, using the same independent testing set (Te_data). The results show that iSuc-ChiDT had a superior overall accuracy (Q^9^ = 0.683) and sensitivity (70.47% vs. 12.20% ~ 37.50%) (Table 11).

**Table 11:** Independent test accuracy for different methods

Method	SN (%)	SP (%)	MCC	Q^9^
SucPred	27.20	67.30	− 0.030	0.436
iSuc-PseAAC	12.20	88.70	0.013	0.374
SuccFind	25.20	79.20	0.029	0.451
SuccinSite	37.10	88.20	0.199	0.548
iSuc-PseOpt	30.30	75.80	0.038	0.478
pSuc-Lys	22.40	82.60	0.036	0.436
Success	14.20	86.80	0.007	0.386
PSuccE	37.50	88.60	0.204	0.551
**iSuc-ChiDT**	**70.47**	**66.27**	**0.205**	**0.683**

Positional information of amino acids is valuable for succinylation site prediction. Most compared methods used binary encoding or physicochemical property encoding to extract positional features. iSuc-ChiDT used chi-square statistical difference table encoding and the experiments showed that it was superior to these two encoding schemes (see Table 8). Moreover, iSuc-ChiDT combined positional features and compositional features to characterize samples. Using the independent tests based on Tr_data and Te_data, the MCC values of 9 positional features, 230 compositional features and 239 combinational features-based models were 0.167, 0.099 and 0.182, respectively, confirming that feature fusion improved predictive accuracy. The ChiDT classifier outperformed traditional classifiers when dealing with imbalanced datasets (see Table 7), further supporting the observation that iSuc-ChiDT could achieve better prediction performance.

## Conclusion

Accurate and rapid prediction of succinylation sites helps researchers to understand the molecular mechanism of succinylation. In this study, we proposed a novel method, iSuc-ChiDT, to identify succinylation sites by incorporating chi-square statistical difference table encoding and the ChiDT classifier. Chi-square statistical difference table encoding is superior to binary encoding and physicochemical property encoding in terms of predictive accuracy and feature dimensions. The ChiDT classifier achieves efficient prediction with a highly imbalanced dataset. iSuc-ChiDT greatly improved sensitivity and overall accuracy compared to previous predictors, and it will serve as an useful complementary tool for detecting potential succinylation sites in proteins. In future studies, we aim to explore more valuable features (e.g. evolutionary information, structural information) for characterizing succinylation sites, in pursuit of better prediction performance.

## Supplementary Information

**Additional file 1: Table S1.** This table shows the 20 × 9 chi-square statistical difference table constructed based on 9 key positions in Tr_data.

**Additional file 2: Table S2.** This table shows 137 rules generated based on Tr_data, and lists the number of positive and negative training samples conforming to each rule.

## Data Availability

All data generated or analyzed during this study are included in this published article and its supplementary information files.

## Abbreviations

ChiDT: chi-square decision table
PTM: post-translational modification
SVM: support vector machine
RF: random forest
ANN: artificial neural network
RVKDE: relaxed variable kernel density estimator
AAC: amino acid composition
PCAAC: pair-coupled amino acid composition
undirected-PCAAC: undirected pair-coupled amino acid composition
SN: sensitivity
SP: specificity
TP: true positive
FP: false positive
TN: true negative
FN: false negative
MCC: Matthews correlation coefficient
MIC: maximal information coefficient
mRMR: minimum redundancy maximum relevance

## References

[1] Zhang ZH, Tan MJ, Xie ZY, Dai LZ, Chen Y, Zhao TM (2011). Identification of lysine succinylation as a new post-translational modification. Nat Chem Biol.

[2] Papanicolaou KN, O’Rourke B, Foster DB (2014). Metabolism leaves its mark on the powerhouse: recent progress in post-translational modifications of lysine in mitochondria. Front Physiol.

[3] Xu XY, Liu T, Yang J, Chen LH, Liu B, Wei CD (2017). The first succinylome profile of Trichophyton rubrum reveals lysine succinylation on proteins involved in various key cellular processes. BMC Genomics.

[4] Shershakova N, Bashkatova E, Babakhin A, Andreev S, Nikonova A, Shilovsky L (2015). Allergen-specific immunotherapy with monomeric allergoid in a mouse model of atopic dermatitis. PLoS ONE.

[5] Tannahill GM, Curtis AM, Adamik J, Palsson-McDermott EM, McGettrick AF, Goel G (2013). Succinate is an inflammatory signal that induces IL-1β through HIF-1α. Nature..

[6] Zhao XW, Ning Q, Chai HT, Ma ZQ (2015). Accurate in silico identification of protein succinylation sites using an iterative semi-supervised learning technique. J Theor Biol.

[7] Hasan MM, Yang SP, Zhou Y, Mollah MN (2016). SuccinSite: a computational tool for the prediction of protein succinylation sites by exploiting the amino acid patterns and properties. Mol BioSyst.

[8] Jia JH, Liu Z, Xiao X, Liu BX, Chou KC (2016). pSuc-Lys: predict lysine succinylation sites in proteins with PseAAC and ensemble random forest approach. J Theor Biol.

[9] Ning Q, Zhao XS, Bao LL, Ma ZQ, Zhao XW (2018). Detecting succinylation sites from protein sequences using ensemble support vector machine. BMC Bioinforma.

[10] Xu Y, Ding YX, Ding J, Lei YH, Wu LY, Deng NY (2015). iSuc-PseAAC: predicting lysine succinylation in proteins by incorporating peptide position-specific propensity. Sci Rep.

[11] Xu HD, Shi SP, Wen PP, Qiu JD (2015). SuccFind: a novel succinylation sites online prediction tool via enhanced characteristic strategy. Bioinformatics..

[12] Dehzangi A, López Y, Lal SP, Taherzadeh G, Michaelson J, Sattar A, Tsunoda T, Sharma A (2017). PSSM-Suc: accurately predicting succinylation using position specific scoring matrix into bigram for feature extraction. J Theor Biol.

[13] López Y, Sharma A, Dehzangi A, Lal SP, Taherzadeh G, Sattar A, Tsunoda T (2018). Success: evolutionary and structural properties of amino acids prove effective for succinylation site prediction. BMC Genomics.

[14] Dehzangi A, López Y, Lal SP, Taherzadeh G, Sattar A, Tsunoda T, Sharma A (2018). Improving succinylation prediction accuracy by incorporating the secondary structure via helix, strand and coil, and evolutionary information from profile bigrams. PLoS ONE.

[15] López Y, Dehzangi A, Lal SP, Taherzadeh G, Michaelson J, Sattar A, Tsunoda T, Sharma A (2017). SucStruct: prediction of succinylated lysine residues by using structural properties of amino acids. Anal Biochem.

[16] Kawashima S, Ogata H, Kanehisa M (1999). AAindex: amino acid index database. Nucleic Acids Res.

[17] Weiss GM, Provost F (2001). The effect of class distribution on classifier learning: An empirical study. Technical Report ML-TR-44. Department of Computer Science, Rutgers University.

[18] Li YT, Dai ZJ, Cao D, Luo F, Chen Y, Yuan ZM (2020). Chi-MIC-share: a new feature selection algorithm for quantitative structure-activity relationship models. RSC Adv.

[19] UniProt Consortium (2011). Ongoing and future developments at the universal protein resource. Nucleic Acids Res.

[20] NCBI protein sequence database. https://www.ncbi.nlm.nih.gov/protein/. Accessed 21 May 2021.

[21] Li WZ, Godzik A (2006). Cd-hit: a fast program for clustering and comparing large sets of protein or nucleotide sequences. Bioinformatics..

[22] PSuccE. https://github.com/ningq669/PSuccE. Accessed 17 April 2021.

[23] Reshef DN, Reshef YA, Finucane HK, Grossman SR, McVean G, Turnbaugh PJ, Lander ES, Mitzenmacher M, Sabeti PC (2011). Detecting novel associations in large data sets. Science..

[24] Reshef DN, Reshef YA, Finucane HK, Grossman SR, McVean G, Turnbaugh PJ, Lander ES, Mitzenmacher M, Sabeti PC (2011). Supporting online material for detecting novel associations in large data sets. Science..

[25] Chen Y, Zeng Y, Luo F, Yuan ZM (2016). A new algorithm to optimize maximal information coefficient. PLoS ONE.

[26] Zeng Y, Yuan HJ, Yuan ZM, Chen Y (2019). A high-performance approach for predicting donor splice sites based on short window size and imbalanced large samples. Biol Direct.

[27] Chou KC (1999). Using pair-coupled amino acid composition to predict protein secondary structure content. J Protein Chem.

[28] Peng H, Long F, Ding C (2005). Feature selection based on mutual information criteria of max-dependency, max-relevance, and min-redundancy. IEEE Trans Pattern Anal Mach Intell.

[29] Sun YM, Kamel MS, Wong AKC, Wang Y (2007). Cost-sensitive boosting for classification of imbalanced data. Pattern Recogn.

[30] Zhang CT, Zhang R (2002). Evaluation of gene-finding algorithms by a content-balancing accuracy index. J Biomol Struct Dyn.

[31] Zhang CT, Ren Z (2003). Q9, a content-balancing accuracy index to evaluate algorithms of protein secondary structure prediction. Int J Biochem Cell Biol.

[32] Zhang QW, Peng QK, Zhang Q, Yan YH, Li KK, Li J (2010). Splice sites prediction of human genome using length-variable Markov model and feature selection. Expert Syst Appl.

[33] Wei D, Zhang HL, Wei YJ, Jiang QS (2013). A novel splice site prediction method using support vector machine. J Comput Inf Syst.

[34] Oyang YJ, Hwang SC, Ou YY, Chen CY, Chen ZW (2005). Data classification with radial basis function networks based on a novel kernel density estimation algorithm. IEEE Trans Neural Netw.

[35] Jia JH, Liu Z, Xiao X, Liu BX, Chou KC (2016). iSuc-PseOpt: identifying lysine succinylation sites in proteins by incorporating sequence-coupling effects into pseudo components and optimizing imbalanced training dataset. Anal Biochem.

